# Gender-specific profiles of tobacco use among non-institutionalized people with serious mental illness

**DOI:** 10.1186/1471-244X-10-101

**Published:** 2010-11-30

**Authors:** Joy L Johnson, Pamela A Ratner, Leslie A Malchy, Chizimuzo TC Okoli, Ric M Procyshyn, Joan L Bottorff, Marlee Groening, Annette Schultz, Marg Osborne

**Affiliations:** 1 School of Nursing, University of British Columbia, T201 - 2211 Wesbrook Mall, Vancouver, BC, Canada V6T 2B5; 2 BC Centre of Excellence for Women's Health, E311 - 4500 Oak Street, Box 48, Vancouver, BC, Canada V6H 3N1; 3 BC Mental Health & Addictions Research Institute, A3-113, 938 W. 28th Avenue, Vancouver, BC, Canada V5Z 4H4; 4 Institute for Healthy Living and Chronic Disease Prevention, University of British Columbia Okanagan, 3333 University Way, Kelowna, BC Canada, V1V 1V7; 5 Cancer Nursing Research, Faculty of Nursing, University of Manitoba, Canada, Room 487 Helen Glass Centre for Nursing, 89 Curry Place, Winnipeg, Manitoba, R3T 2N2

## Abstract

**Background:**

In many countries, smoking remains the leading preventable cause of death. In North America, reductions in population smoking levels are stabilising and, in recent years, those involved in tobacco control programming have turned their attention to particular segments of society that are at greatest risk for tobacco use. One such group is people with mental illness. A picture of tobacco use patterns among those with mental illness is beginning to emerge; however, there are several unanswered questions. In particular, most studies have been limited to particular in-patient groups. In addition, while it is recognised that men and women differ in relation to their reasons for smoking, levels of addiction to nicotine, and difficulties with cessation, these sex and gender differences have not been fully explored in psychiatric populations.

**Methods:**

Community residents with serious mental illness were surveyed to describe their patterns of tobacco use and to develop a gender-specific profile of their smoking status and its predictors.

**Results:**

Of 729 respondents, almost one half (46.8%) were current tobacco users with high nicotine dependence levels. They spent a majority of their income on tobacco, and reported using smoking to cope with their psychiatric symptoms. Current smokers, compared with non-smokers, were more likely to be: diagnosed with a schizophrenia spectrum disorder (rather than a mood disorder); male; relatively young; not a member of a racialised group (e.g., Aboriginal, Asian, South Asian, Black); poorly educated; separated or divorced; housed in a residential facility, shelter, or on the street; receiving social assistance; and reporting co-morbid substance use. There is evidence of a gender interaction with these factors; in the gender-specific multivariate logistic regression models, schizophrenia spectrum disorder versus mood disorder was not predictive of women's smoking, nor was education, marital status or cocaine use. Women, and not men, however, were more likely to be smokers if they were young and living in a residential facility.

**Conclusion:**

For men only, the presence of schizophrenia spectrum disorder is a risk factor for tobacco use. Other factors, of a social nature, contribute to the risk of smoking for both men and women with serious mental illness. The findings suggest that important social determinants of smoking are "gendered" in this population, thus tobacco control and smoking cessation programming should be gender sensitive.

## Background

In many countries, smoking remains the leading preventable cause of death. In North America, reductions in smoking rates are stabilising and, in recent years, those involved in tobacco control programming have turned their attention to particular segments of society that are at greatest risk for tobacco use, especially people with mental illness.

An appreciation of the high rate of tobacco use by those with mental illness is emerging. In a USA population-based study of 4,441 respondents aged 15-54 years, Lasser and colleagues [1] reported that current smoking rates for those with no mental illness, lifetime mental illness, or mental illness in the past month were 22.5%, 34.8%, and 41.0%, respectively. The burden of tobacco use appears to be disproportionally borne by those with mental illness. Dani and Harris reported that 7% of Americans have a mental illness, and that this relatively small group consumes 34% of all cigarettes sold in the USA [2]. Those with mental illness are noted to have a higher "all cause" mortality rate compared with the general population; although suicide and accidents contribute to the high rate, very high mortality rates due to cardiovascular disease are apparent [3].

Those with serious mental illness (SMI) (i.e., those individuals who require long-term treatment for their illness) are at particular risk for tobacco use. Previous studies have found very high smoking rates among selected populations of people with SMI, including psychiatric outpatients [4], patients in state mental hospitals in the USA, and patients in several other countries [5,6]. There is some evidence that smoking rates vary by psychiatric diagnosis, with individuals with a diagnosis of schizophrenia having the highest tobacco use rate [7].

Sex and gender differences in tobacco use have been the focus of numerous studies. It is increasingly recognised that men and women differ in relation to their reasons for smoking, levels of addiction to nicotine, and difficulties with cessation. Some of these differences may be attributed to social factors (gender) while others may be attributable to biological factors (sex) [8]. These sex and gender differences have not been fully explored in psychiatric populations.

Although it is now recognised that substance use disorders are prevalent among people with SMI, tobacco use is often not included in substance use screening [9], even though there are emerging links being made between tobacco use and other substance use and in some instances with antipsychotic medication use [10]. There is limited understanding of whether those with SMI who use tobacco are also more likely to use other substances, and if so, which substances are most frequently used.

A picture of tobacco use patterns among those with SMI is emerging; however, there are several unanswered questions. In particular, much of the data collected have been limited to particular clinics or in-patient groups, and few researchers have disaggregated their data by gender. Given recent trends of deinstitutionalisation, further study is warranted of tobacco use patterns among men and women living in the community with SMI. There also is a need to explore how tobacco use varies by diagnosis, whether it differs by symptomatology and other substance use, and whether social-environmental factors are salient.

The purpose of this study was to determine the rate of tobacco use among people with SMI accessing community-based mental health services, and to learn more about the factors associated with their tobacco use. The specific objectives of the research were to: (a) describe the profile of tobacco use among people with SMI, (b) determine whether tobacco use differs by psychiatric diagnosis and by gender, and (c) determine the extent to which co-morbid substance use and social-environmental factors are associated with smoking status.

## Methods

We conducted a cross-sectional survey in which we targeted all adults with SMI who received services from community-based mental health teams in Vancouver, Canada. The vast majority of non-institutionalised persons with a diagnosis of SMI, in this city, are followed by one of these teams (they provide services to almost 6,000 people, more than 1% of Vancouver's population). Each mental health team provides psychiatric assessment and comprehensive treatment through drop-in and outreach services for people in their catchment area. Services include medication management, individual and group therapy, rehabilitation, and education. Many clients receive additional support in the form of rehabilitation programming or housing through contracted agencies.

### Sample

We sought to obtain a representative sample of people with SMI receiving community mental health services. Because of confidentiality concerns (i.e., disclosure of names and diagnoses without consent), however, we were not permitted to draw a random sample from the population of people receiving services. Consequently, we recruited voluntary participants who were receiving services from seven of the eight mental health teams. Eligible participants were individuals whose health records were flagged as active and who received care from an adult care program. All study participants were living in the community and were able to communicate and be understood in English, Mandarin, Cantonese, or Punjabi.

### Procedures

The research staff visited each community mental health team, provided information about the study, answered questions, and negotiated strategies to access eligible participants. A research assistant recruited participants at the mental health team offices during regular operating hours. The participants were introduced to the survey either through the reception desk personnel or their case managers. The participants could "self refer" to the research staff in response to brochures and flyers available in the office waiting areas. The research staff explained the study in detail, obtained written, fully informed consent, and administered the questionnaire [11]. Upon completion of the questionnaire, the participants received a $10 gift certificate for a local grocery store. Data collection occurred between October 2005 and October 2006, with each mental health team involved for approximately 4-6 months.

### Ethical approval

Ethical approval was obtained from the Behavioural Research Ethics Board of the University of British Columbia. Approval to conduct the research was obtained from Vancouver Coastal Health, Vancouver Community Health Service Delivery Area.

### Measures

The questionnaire, which included several scales and items, requiring 20-45 minutes to complete, was administered by the research staff.

#### Demographics

The demographic items included: age ("What is your birth date?"), gender ("Do you identify as male, female, trans-gendered or other?"), and ethnic/cultural background ("What would you say is your main ethnic or cultural background?"). The information from this item was used to create a "racialised group" variable ("no" or "yes"). The use of this term is meant to construe the belief that racial classifications are socially constructed and embedded in Eurocentric notions of inferiority, colonization, and prestige [12]. In the study community, people who are Aboriginal, Asian, South Asian or Black tend to be racialised, which has implications for their health [13]. The other demographic variables included: marital status ("What is your current marital status?"), current living situation ("Who do you live with? Alone, with family, friend(s), group home, or other?"), and housing type ("What kind of housing do you live in?" Independent, semi-independent, residential, shelter/hostel, no fixed address, other?), financial support ("In the last month, where have you received money or financial support from? Earned income/paid work, social assistance/welfare, disability benefits, unemployment insurance, pension, savings, alimony/child support, family contribution, panhandling, other"), disposable income ("After paying for housing and food last month, how much money did you have to spend on yourself?"), and income "prioritizing strategies" ("When you have to make decisions about spending money on cigarettes, have you ever chosen to give up anything so that you would have enough tobacco? Have you given up buying food? Coffee? Bus fare? Rent? Medication? Anything else?").

#### Psychiatric Diagnosis

Not all of the participants (15.1%) provided permission to access their medical records. These individuals' diagnostic information was limited to a self-report of the psychiatric diagnosis ("What is your diagnosis?"). For the remainder who provided consent (84.9%), information about their diagnoses was collected from their existing mental health team medical record. Once referred to a community mental health team, all clients are assessed by one of the team's psychiatrists. The psychiatrists typically base their diagnoses on findings of a one-hour assessment interview (that includes mental status examination and case history). DSM IV criteria are used to guide the diagnostic process. A diagnosis is recorded at the time of the client's intake to community mental health services, and then modified as required. For the purposes of this study, the most current diagnosis was recorded.

For the purpose of the analysis, we classified the specific diagnoses as schizophrenia spectrum disorders, mood disorders, or anxiety disorders. A diagnosis of a *schizophrenia spectrum disorder *included schizophrenia and its subtypes, schizoaffective disorder, delusional disorder, or psychosis not otherwise specified. *Mood disorders *included diagnoses of bipolar disorder, major depression, manic depression or dysthymia. *Anxiety disorders *included diagnoses of obsessive compulsive disorder, generalized anxiety disorder, and panic disorder.

#### Psychiatric Symptoms

Psychiatric symptoms were assessed with the Brief Symptom Inventory (BSI) [14], which has been validated for use with people living with schizophrenia and is preferred over other scales of psychopathology because it is relatively non-invasive, quick to administer, and suitable for use by research staff [15]. The 18-item scale measures anxiety (e.g., nervousness or shakiness inside), depression (e.g., feeling lonely), and general somatic symptoms (e.g., feeling weak in parts of your body) using a 5-point scale to measure the extent of distress experienced over the past week; the response options were: "not at all," "a little bit," "moderately," "quite a bit," and "extremely." The internal consistency for the Global Severity Index (GSI) has been reported to be strong with a coefficient alpha of .89 [15]. In this study, the scale had a coefficient alpha of .92. We followed the prescribed BSI scoring method: the *raw *GSI score was calculated by adding the 18 items [16]. If participants had more than 2 item responses missing for any subscale, their scores were not calculated and the case was treated as missing. When participants had 1 or 2 missing items, values were imputed by rounding the mean of the completed items to the nearest whole number. The GSI scores were standardized using T scores with a mean of 50 and an SD of 10 to determine "caseness." Those with GSI scores of 63 or greater were deemed to be at positive risk for psychological distress [14,16].

#### Tobacco Use Patterns

Smoking status was determined by asking the participants if they had "ever" smoked, whether they had smoked more than 100 cigarettes in their lifetime, when they smoked their last cigarette, and if they smoked every day [17]. The participants were classified as non-smokers (had never smoked or smoked less than 100 cigarettes), former smokers (had smoked more than 100 cigarettes, but had not smoked in the past 30 days), or current smokers (had smoked more than 100 cigarettes and had smoked in the past 30 days). A binary variable was created with current smoker versus former/never smoker. The participants also were asked, "Do you consider yourself a current smoker?" (The response options were "yes" or "no.") There was excellent agreement between the classification of smoking status based on the number of cigarettes smoked in the past 30 days and the participants' self-reported smoking status (Kappa = .97).

Tobacco use patterns and practices were measured by determining the amount of tobacco smoked each day, the age of smoking initiation [18] and reasons for tobacco use [19]. Physical health consequences of tobacco use were assessed with the item, "Do you have, or have you had symptoms that you believe were caused or made worse by smoking?" [20]. Items also were included to determine: the primary sources of tobacco procurement ("As you know, cigarettes are expensive and people get them in different ways. Where do you get yours?"), average weekly expenditure on tobacco ("About how much money do you spend on tobacco per week?"), and type of cigarettes smoked ("What kind of cigarettes do you smoke... store bought, roll your own, butts, other?").

Nicotine dependence was measured with the Fagerström Test for Nicotine Dependence (FTND) [20]. This test is appropriate for the assessment of nicotine dependence in smokers with schizophrenia [21]. The coding algorithm yields a total score of 0-10. Scores above 6 are indicative of a high level of dependence. Although widely used, the internal consistency for the FTND scale has been borderline (Cronbach's alpha .67) [22]; in this study, the Cronbach's alpha was .50. In addition to using this scale, the participants were asked to rate their tobacco addiction using a self-rated addiction scale of 0-10, where 0 was "not at all" addicted and 10 was "extremely" addicted. They also were asked about using tobacco to manage their psychiatric symptoms: "Some people use smoking to cope with their symptoms, such as having anxiety or hearing voices. How often do you smoke to cope with symptoms?" The item was scored with a 4-point scale rated as "not at all," "a little," "somewhat," or "a great deal." Another open-ended question asked, "What symptoms do cigarettes help you manage?"

#### Substance Use

Comorbid substance use was assessed with items from the substance use section of the Addiction Severity Index (ASI), originally developed for clinical purposes [23], [24]. The ASI has seven sections measuring various aspects of an individual's life that may be affected by substance use. For research purposes, the use of individual items from the substance use section of the ASI has been found to be reliable, valid, and valuable [25]. The participants were asked, "How many days in the past month (last 30 days) did you use...any alcohol? Alcohol to get drunk? Heroin (smack, junk)? Methadone? Opium, codeine, or pain killers like Tylenol 3? Sedatives, hypnotics or tranquilizers like Valium or Xanax? Cocaine or crack? Amphetamines, like speed, E or meth? Marijuana (weed, pot)? Hallucinogens, like LSD or mushrooms? Inhalants, like glue, paint thinner or gas? Any other substances? Specify." The ASI results were reported as number of days and were categorized into "no, none" or "yes, 1 or more days" because of the participants' infrequent regular use and the distributional properties of their responses [26].

### Analysis

A total of 788 people participated in the study, which represents approximately 20% of the clients who received care from the 7 community mental health teams. The data from these clients were cleaned and screened before analysis to ensure missing data were random in occurrence and that all data were within their excepted ranges. Responses from 59 (7.5%) individuals were excluded because they did not have a clear psychiatric diagnosis. Descriptive analysis of the sample (N = 729) employed chi square tests to determine the associations between psychiatric diagnosis and the categorical study variables. Independent sample *t*-tests employing Levine's test for equality of variance were employed to examine the relationships between psychiatric diagnosis and the continuous variables. We employed Hosmer and Lemeshow's model-building process to determine the variables that were associated with current smoking status (current smoker vs. former/never smoker) [27]. First, we employed univariate logistic regression analyses to identify the study variables associated with smoking status and conducted these analyses for the entire sample and for men and women, separately. In the second step, variables that were associated with smoking status at *p *≤ .25 were included in the multivariate logistic regression models (all participants and gender-specific). To obtain the most parsimonious and stable models, we then trimmed them by removing statistically non-significant variables sequentially by examining the Wald statistic and comparison of the likelihood ratios. If the likelihood ratio test was significant when a non-significant variable was removed (i.e., *p *< .05), then the variable was added back to the model. Once the main effects models were finalized, all possible interactions between diagnostic category and the other variables were examined. All analyses were conducted with IBM SPSS Statistics 18.

## Results

### Demographics

About one half (51.2%) of the participants were women; 26.6% were of a racialised group; 76.5% had a high school or better education; 63.0% reported being single and never married; 71.0% lived in independent, private houses or apartments; 52.9% lived alone; and the majority (56.7%) received government disability benefits. The average age of the participants was 47.4 years (SD = 12.1) (see Table 1). To determine if those who provided access to their records differed from those who did not, we compared the two groups by the variables listed in Table 1 and found no statistically significant differences.

**Table 1:** Demographic Characteristics and Participants' Substance Use by Diagnostic Category

Characteristic	All		Schizophrenia Spectrum Disorder		**Mood or Anxiety Disorder**^**1**^		Differences
	(N = 729)		(n = 436)		(n = 293)		
	(100%)		(59.8%)		(40.2%)		
	
	f	%	f	%	f	%	**χ**^**2 **^**(df), sig.**^**2**^
**Gender (n = 719)**							8.0 (1), p = .005
Male	351	48.8	228	53.3	123	42.3	
Female	368	51.2	200	46.7	168	57.7	
**Racialised Group (n = 680)**							0.3 (1), p = .592
No (e.g., white/European)	499	73.4	300	74.3	199	72.1	
Yes (e.g., Aboriginal/Asian/South Asian/Black)	181	26.6	104	25.7	77	27.9	
**Education (n = 723)**							1.1 (1), p = .289
Less than high school	170	23.5	108	25.0	62	21.3	
High school or more	553	76.5	324	75.0	229	78.7	
**Marital Status (n = 719)**							18.9 (3), p = <.001
Single and never married	453	63.0	289	67.5	164	56.4	
Separated/Divorced	159	22.1	92	21.5	67	23.0	
Married (spouse or common law partner)	79	11.0	30	7.0	49	16.8	
Widowed	28	3.9	17	4.0	11	3.8	
**Housing (n = 723)**							28.5 (3), p <.0001
Independent (private house or apartment)	513	71.0	279	64.6	234	80.4	
Residential facility (licensed/boarding)	102	14.1	81	18.8	21	7.2	
Semi-independent (subsidy/supportive care)	94	13.0	66	15.3	28	9.6	
Shelter/hostel/no housing	14	1.9	6	1.4	8	2.7	
**Living Arrangement (n = 724)**							26.4 (3), p <.0001
Lives alone	383	52.9	240	55.6	143	49.0	
Lives with family	170	23.5	87	20.1	83	28.4	
Group home resident	101	14.0	76	17.6	25	8.6	
Lives with roommate/friend(s)/girlfriend/boyfriend	70	9.7	29	6.7	41	14.0	
**Sources of Financial Support (multiple responses permitted, n = 714)**							
Disability benefits (yes v. no)	405	57.0	235	55.8	170	58.8	0.7 (1), p = .397
Canada Pension Plan or other pension (yes v. no)	165	23.1	102	24.1	63	21.7	0.4 (1), p = .525
Earned income/paid work (yes v. no)	167	23.4	89	21.0	78	26.9	3.0 (1), p = .082
Social assistance/welfare (yes v. no)	119	16.7	87	20.5	32	11.0	10.5 (1), p = .001
Family contribution (yes v. no)	110	15.4	58	13.7	52	17.9	2.1 (1), p = .150
**Smoking Status (n = 729)**							13.1 (2), p = .001
Current	341	46.8	226	51.8	115	39.2	
Former	156	21.4	91	20.9	65	22.2	
Never	232	31.8	119	27.3	113	38.6	
**Any Alcohol Intoxication (in past month) (n = 716)**							2.9 (1), p = .088
Yes	63	8.8	31	7.2	32	11.2	
No	653	91.2	399	92.8	254	88.8	
**Any Cocaine Use (in past month) (n = 716)**							0.0 (1), p = 1.000
Yes	28	3.9	17	4.0	11	3.8	
No	688	96.1	412	96.0	276	96.2	
**Any Cannabis Use (in past month) (n = 717)**							2.3 (1), p = .128
Yes	92	12.8	48	11.2	44	15.3	
No	625	87.2	382	88.8	243	84.7	
	
	**Mean**	**SD**	**Mean**	**SD**	**Mean**	**SD**	**t (df), sig**.
	
**Age (years) (n = 721)**	47.4	12.1	47.8	12.4	46.9	11.9	1.0 (719), p = .336
**Brief Symptom Inventory (n = 715)**							
Somatic symptoms	10.8	4.3	10.6	4.1	11.1	4.6	-1.6 (566.1), p = .100^3^
Depression	12.0	5.4	11.5	4.9	12.8	6.0	-3.1 (522.4), p = .002^3^
Anxiety	11.8	5.3	11.3	4.9	12.6	5.8	-3.2 (533.1), p = .002^3^
Global Severity Index	34.7	13.2	33.4	12.1	36.6	14.5	-3.1 (533.6), p = .002^3^

^1 ^Composed of participants with a mood disorder or an anxiety disorder (38.1% and 2.1% of the total sample, respectively).

^2^Continuity correction applied for crosstabulations with 1 degree of freedom.

^3^Levene's Test for Equality of Variances significant; thus, equal variances not assumed for t-tests.

### Psychiatric Diagnostic Category

The majority (59.8%) of the participants had a diagnosis of schizophrenia spectrum disorder and the remainder had mood (38.1%) or anxiety (2.1%) disorders. For the subsequent analyses, we combined those with a mood disorder or anxiety disorder into a single group. The participants with schizophrenia spectrum disorder were more likely to be male, single and never married, live in a residential facility or group residential home, and receive social assistance (see Table 1).

The mean BSI scores for the sample were: somatisation = 10.8 (SD = 4.3), depression = 12.0 (SD = 5.4), and anxiety = 11.8 (SD = 5.3) (see Table 1). In terms of 'caseness' of psychological distress, 12.2% of the participants surpassed the GSI cutoff value of 63 or greater. In general, those with mood or anxiety disorders had greater symptomatology; 15.4% of this group, compared with 10.0% of those with schizophrenia spectrum disorder met the 'caseness' criterion.

### Tobacco Use

Almost one half (46.8%) of the participants were current smokers (see Table 1); 57.5% of the men and 35.6% of the women were current smokers. The prevalence of participants who reported "ever smoking" was 89.3%. Most (53.8%) of the participants began smoking at 15 years of age or younger. Of those who currently smoked, the average number of cigarettes smoked daily was 20.2 cigarettes (SD = 13.9), and the main reasons reported for smoking were addiction (36.8%) and anxiety (37.1%). The majority of current smokers reported smoking every day (96.2%), had smoked for 30 years, on average, and were self-identified "chain smokers" (61.5%). Almost one third of the current smokers reported lighting a second cigarette while the first cigarette was still burning (27.4%). The current smokers' median FTND score was 6.0. In relation to their self-rated addiction, the mean response was = 7.4 (SD = 2.5) on a scale of 0 to 10. Although the self-rated addiction scores were not significantly associated with the FTND scores (Spearman rho = .03, *p *= .70), they were associated with the average number of cigarettes smoked per day (Spearman rho = .44, *p *< .001) and age of smoking initiation (Spearman rho = -.12, *p *= .030). About one half (51.5%) of the participants revealed that they had experienced symptoms of a disease or illness that were caused or worsened by their smoking.

Almost all (92.2%) of the current smokers reported "buying tobacco from a store," which was the most common method of procuring tobacco, although it was not exclusive to other methods including "receiving tobacco from friends" (53.3%), "bumming cigarettes from people" (39.6%), "sharing someone else's" (39.5%), and "picking up butts" (30.5%) (i.e., picking up cigarette ends from sidewalks and ashtrays and smoking the ends or re-rolling the salvaged tobacco). The average amount of money spent per week on tobacco was (CAD) $40.50 (SD = $25.70). Almost one half (41.2%) of the current smokers indicated that they had, on occasion, given up buying food so that they would have enough tobacco.

Many of the current smokers (68.8%) reported that they coped with their psychiatric symptoms by smoking and 30.3% reported doing this "a great deal." Those who answered affirmatively indicated that cigarettes helped them manage multiple symptoms including anxiety/stress (95.9%), depression (20.6%), and hearing voices/delusions (10.0%).

### Bivariate associations with current smoking status

The men with a schizophrenia spectrum disorder, in the sample, were 1.8 times more likely to be current smokers than were those men with a mood or anxiety disorder (see Table 1). The association between diagnostic category and smoking status was not significant for the women. The GSI score (≥ 63 vs. < 63; 'caseness') was not statistically significantly associated with smoking (see Table 2).

**Table 2:** Bivariate Relationships between Smoking Status (Current vs. Former/Never) and Diagnostic Category, Demographic Characteristics and Other Substance Use

Characteristic	All		Men		Women	
	
	Odds Ratio	95% CI	Odds Ratio	95% CI	Odds Ratio	95% CI
**Diagnostic Category**^1^						
Schizophrenia spectrum disorder	1.7**	1.2 - 2.3	1.8**	1.2 - 2.8	1.3	0.8 - 1.9
Mood or anxiety disorder (referent)	1.0	--	1.0	--	1.0	--
**Gender**						
Men	2.5***	1.8 - 3.3	--	--	--	--
Women (referent)	1.0	--	--	--	--	--
**Age Group**						
17-29 years	2.4*	1.2 - 4.7	1.6	0.6 - 4.3	2.8*	1.1 - 7.0
30-49 years	1.7*	1.1 - 2.6	1.8	0.9 - 3.7	1.1	0.6 - 2.0
50-59 years	2.3**	1.4 - 3.7	2.4*	1.1 - 5.1	1.8	0.9 - 3.4
60+ years (referent)	1.0	--	1.0	--	1.0	--
**Racialised Group**						
No (e.g., white/European)	1.8**	1.3 - 2.6	1.4	0.9 - 2.4	2.4**	1.4 - 4.1
Yes (e.g., Aboriginal/Asian/South Asian/Black) (referent)	1.0	--	1.0	--	1.0	--
**Education**						
Less than high school	1.8**	1.3 - 2.6	2.1**	1.3 - 3.4	1.2	0.7 - 2.1
High school or more (referent)	1.0	--	1.0	--	1.0	--
**Marital Status**						
Single and never married	1.6	1.0 - 2.7	2.0	0.8 - 4.8	0.9	0.5 - 1.7
Separated/Divorced	2.0*	1.1 - 3.5	3.3*	1.2 - 8.9	1.4	0.7 - 2.8
Widowed	1.2	0.5 - 2.9	4.7	0.4 - 52.1	1.0	0.4 - 2.7
Married (spouse or common law partner) (referent)	1.0	--	1.0	--	1.0	--
**Housing**						
Independent (private house or apartment) (referent)	1.0	--	1.0	--	1.0	--
Residential facility (licensed/boarding)	2.0**	1.3 - 3.0	1.4	0.7 - 2.5	2.7**	1.4 - 5.1
Semi-independent (subsidy/supportive care)	1.4	0.9 - 2.1	1.2	0.7 - 2.3	1.5	0.8 - 2.9
Shelter/hostel/no housing^2^	17.9**	2.3 -137.7	M	--	M	--
**Living Arrangement**						
Lives alone	2.2***	1.5 - 3.1	2.1*	1.2 - 3.6	1.9*	1.1 - 3.3
Lives with family (referent)	1.0	--	1.0	--	1.0	--
Group home resident	3.4***	2.0 - 5.6	2.4*	1.1 - 5.1	4.3***	2.1 - 8.8
Lives with roommate/friend(s)/girlfriend/boyfriend	2.1*	1.2 - 3.7	2.7*	1.1 - 6.4	1.6	0.7 - 3.6
**Sources of Financial Support (multiple responses permitted)**						
Disability benefits (yes v. no)	0.9	0.6 - 1.1	0.8	0.5 - 1.2	0.8	0.5 - 1.3
Canada Pension Plan or other pension (yes v. no)	0.7	0.5 - 1.1	0.6	0.4 - 1.0	1.0	0.6 - 1.6
Earned income/paid work (yes v. no)	0.8	0.5 - 1.1	0.7	0.4 - 1.1	0.8	0.5 - 1.3
Social assistance/welfare (yes v. no)	3.3***	2.1 - 5.0	3.1***	1.7 - 5.8	3.2***	1.7 - 5.9
Family contribution (yes v. no)	0.8	0.5 - 1.2	0.9	0.4 - 1.9	1.0	0.6 - 1.6
**Any Alcohol Intoxication (in past month)**						
Yes	2.2**	1.3 - 3.7	2.1*	1.0 - 4.2	1.4	0.5 - 3.5
No (referent)	1.0	--	1.0	--	1.0	--
**Any Cocaine Use (in past month)**						
Yes	7.5***	2.6 - 21.8	8.4**	1.9 - 36.3	3.7	0.9 - 20.6
No (referent)	1.0	--	1.0	--	1.0	--
**Any Cannabis Use (in past month)**						
Yes	5.5***	3.2 - 9.3	4.6***	2.0 - 10.5	5.2***	2.5 - 10.5
No (referent)	1.0	--	1.0	--	1.0	--
**Brief Symptom Inventory (Global Severity Index)**						
< 63 (referent)	1.0	--	1.0	--	1.0	--
≥ 63	1.3	0.8-2.0	1.0	0.5 - 2.0	1.6	0.8 - 3.0

^1 ^76 (38.0%) of the 200 women with schizophrenia spectrum disorders were current smokers. 55 (32.7%) of the 168 women with mood or anxiety disorders were current smokers. 143 (62.7%) of the 228 men with schizophrenia spectrum disorders were current smokers. 59 (48%) of the 123 men with a mood or anxiety disorders were current smokers.

^2^Treated as missing in gender-specific models because of small numbers.

*p < .05; **p < .01; ***p < .001.

The men were 2.5 times more likely to smoke than were the women (see Table 2). For women, being young was a risk factor (those 17-29 years of age were 2.8 times were more likely to smoke compared with those 60+ years of age). For men, the age group with the greatest risk of smoking was the 50-59 years of age group (OR = 2.4, 95% CI: 1.1-5.1).

Being a member of racialised group was protective against smoking for the women only. White/European women were 2.4 times more likely to smoke compared with racialised women. Education was only significant for the men; those with less than high school education were about twice as likely to smoke compared with those who were better educated. Compared with those who were married, men who were separated or divorced were 3.3 times more likely to smoke. Marital status and education were not risk factors for the women.

The respondents who reported having no housing or who lived in temporary shelters or hostels were very likely to smoke (OR = 17.9; 95% CI: 2.3-13.7). There were too few cases of people without housing to provide a breakdown by gender. Other forms of housing, however, also placed the women at risk of smoking; specifically, women in residential facilities were 2.7 times more likely to smoke than were women who lived independently. Similarly, living with their family protected both men and women from smoking (see Table 2).

The only form of financial support received that was associated with smoking status was social assistance or welfare. Both men and women who received this form of support were thrice as likely to smoke compared with those not on assistance.

Other substance use was associated with smoking status. For men who used alcohol to intoxication in the previous month, or who had used any cocaine or cannabis in the past month, current tobacco smoking was also likely. For women, the only other substance use that was associated with their smoking status was cannabis use (OR = 5.2; 95% CI: 2.5-10.5) (see Table 2).

### Multivariate associations with current smoking status

The multivariate gender-specific models revealed the following. For the men, the significant predictors of smoking status, adjusted for confounding, were: having a schizophrenia spectrum disorder vs. a mood or anxiety disorder (OR_adjusted _= 2.0; 95% CI: 1.2-3.3), having less than a high school education (OR_adjusted _= 1.8; 95% CI: 1.0-3.1), being separated or divorced, rather than married (OR_adjusted _= 3.8; 95% CI: 1.2-11.4), receiving social assistance or welfare (OR_adjusted _= 2.6; 95% CI: 1.3-5.4), and having used cannabis in the past month (OR_adjusted _= 4.6; 95% CI: 2.2-10.0) (see Table 3). Being a member of a racialised group and having used cocaine in the past month had odds ratios that spanned unity; retaining these variables in the model, however, improved the model (the comparison of log-likelihood ratios for models with and without these variables were statistically significant). The Nagelkerke *R*^*2 *^for this model, with seven variables, was .23. The correct classification rates were 63.8% for current smokers and 70.9% for non-smokers; the overall correct classification rate was 67.0%.

**Table 3:** Multivariate Relationships between Smoking Status (Current vs. Former/Never) and Diagnostic Category, Demographic Characteristics and Other Substance Use

Characteristic	All		Men		Women	
	
	Adjusted Odds Ratio	95% CI	Adjusted Odds Ratio	95% CI	Adjusted Odds Ratio	95% CI
**Diagnostic Category**						
Schizophrenia spectrum disorder	1.5*	1.0 - 2.1	2.0*	1.2 - 3.3	NI^1^	--
Mood or anxiety disorder (referent)	1.0	--	1.0	--	--	--
**Gender**						
Men	2.0***	1.4 - 2.9	--	--	--	--
Women (referent)	1.0	--	--	--	--	--
**Age Group**						
17-29 years	2.6*	1.2 - 5.8	NI	--	2.8*	1.0 - 8.0
30-49 years	1.4	0.8 - 2.5	--	--	1.0	0.5 - 2.0
50-59 years	1.8*	1.0 - 3.1	--	--	1.7	0.9 - 3.5
60+ years (referent)	1.0	--			1.0	--
**Racialised Group**						
No (e.g., white/European)	1.8**	1.2 - 2.7	1.5	0.8 - 2.6	2.5**	1.4 - 4.6
Yes (e.g., Aboriginal/Asian/South Asian/Black) (referent)	1.0	--	1.0	--	1.0	--
**Education**						
Less than high school	--	--	1.8*	1.0 - 3.1	NI	--
High school or more (referent)	--	--	1.0	--	--	--
**Marital Status**^2^						
Single and never married	1.0	0.5 - 1.7	1.7	0.6 - 4.4	NI	--
Separated/Divorced	1.8	1.0 - 3.5	3.8*	1.2 - 11.4	--	--
Widowed	1.4	0.5 - 4.1	C	--	--	--
Married (spouse or common law partner) (referent)	1.0	--	1.0	--	--	--
**Housing**						
Independent (private house or apartment) (referent)	1.0	--	NI	--	1.0	--
Residential facility (licensed/boarding)	1.8*	1.1 - 3.1	--	--	2.7**	1.3 - 5.8
Semi-independent (subsidy/supportive care)	1.6	1.0 - 2.6	--	--	1.9	0.9 - 3.8
Shelter/hostel/no housing	M^3^	--	--	--	M	--
**Source of Financial Support**						
Social assistance/welfare (yes v. no)	2.7***	1.6 - 4.4	2.6*	1.3 - 5.4	3.3***	1.6 - 6.5
**Any Cocaine Use (in past month)**						
Yes	--	--	4.9	1.0 - 24.0	--	--
No (referent)	--	--	1.0	--	--	--
**Any Cannabis Use (in past month)**						
Yes	4.5***	2.5 - 8.1	4.6***	2.2 - 10.0	3.2*	1.2 - 8.0
No (referent)	1.0	--	1.0	--	1.0	--

^1 ^Not included in the model because the bivariate relationship (unadjusted odds ratio) had a *p *value ≥ .25 (NI).

^2^Widowed combined with separated/divorced in gender-specific models because of small numbers (C).

^3^Treated as missing in gender-specific models because of small numbers (M).

*p < .05; **p < .01; ***p < .001.

For the women, the significant predictors of smoking status were: age (17-29 years vs. 60+ years; OR_adjusted _= 2.8; 95% CI: 1.0-8.0), being white or of European origin (OR_adjusted _= 2.5; 95% CI: 1.4-4.6), living in a residential facility vs. independent living (OR_adjusted _= 2.7; 95% CI: 1.3-5.8), receiving social assistance or welfare (OR_adjusted _= 3.3; 95% CI: 1.6-6.5), and having used cannabis in the past month (OR_adjusted _= 3.2; 95% CI: 1.2-8.0) (see Table 3). The Nagelkerke *R*^*2 *^for this model, with five variables, was .17. The correct classification rates were 37.6% for current smokers and 86.9% for non-smokers; the overall correct classification rate was 69.5%.

## Discussion

It is noteworthy that almost one half of the study participants were current smokers; this is almost three times the 2007 smoking rate of 14% in the province of British Columbia, Canada [28]. The participants tended to be heavy smokers who were highly dependent on nicotine. Other researchers also have reported very high rates of tobacco dependence among people with serious mental illness [6], particularly those with schizophrenia [29]. What is particularly troubling about our findings is that Vancouver is a region that has some of the strongest tobacco control measures in Canada [30]. Although these measures have been instrumental in reducing the smoking rate to one of the lowest in Canada, a more tailored approach with considerable support, including pharmacological aid, social support and other resources, is needed for community-based people with serious mental illness.

We found that tobacco use rates varied by psychiatric diagnosis (39.2% for those with mood and anxiety disorders and 59.8% for those with schizophrenia), and that diagnosis was only predictive of men's smoking. The overall rate is lower than what has been reported elsewhere. It has been reported that, in Kentucky, the prevalence of current daily smoking for patients with bipolar disorder and schizophrenia were 66% and 74%, respectively [31]. This may point to the importance of the social context in influencing the tobacco use of people with serious mental illness. Kentucky, a tobacco producing state in the USA, is reported to have the highest current smoking prevalence rate in the USA [32].

More men than women reported being current smokers and the predictors of tobacco use varied by gender, in the gender-stratified analysis we found differential predictors of current smoking status. These findings suggest that while strategies need to be found for people with mental health issues, in general, services need to be gender sensitive. Gender has historically been a factor in tobacco use; men have been more likely to smoke than have women. Although the gender gap in the general population's smoking rate is narrowing, there remains a substantial differential in the smoking rates of men and women with serious mental illness. More research is needed of people with serious mental illness to untangle the relationships among gender, psychiatric diagnosis, the social context, and smoking status.

The specific needs of people with a diagnosis of schizophrenia spectrum disorder are unique. For example, they may require more support for cessation and they may need education about how their negative symptoms may interfere with some of the conventional methods of cessation support such as group interaction. The finding that smokers had higher rates of substance use than did the non-smokers echoes the results of other researchers and magnifies the overlap between tobacco use and other substance use. Best practice guidelines recommend that treatment for these co-occurring disorders be integrated [33]. Although movement towards the integration of mental health and addiction services is gaining momentum, and more settings have begun to successfully incorporate smoking cessation into their practice [34], there is still much dispute among clinicians about whether tobacco use should be treated as an addiction and considered part of the spectrum of substance use within the context of dual disorder services.

Many of the smokers in this study reported strategically using tobacco to cope with their psychiatric symptoms. Reports published elsewhere have discussed the complicated roles nicotine and tobacco play in the lives of people with mental illness [35]. The stimulating effect of nicotine is known to modulate social and interpersonal factors to reduce anxiety and to relieve boredom. Nicotine also alters the neurochemistry of the brain and affects the rate at which psychotropic medications are metabolised [35]. Clearly the use of tobacco has serious implications for psychiatric recovery, which is a compelling reason to advocate strongly for the clinical monitoring of changes in tobacco use in clients.

Tobacco cessation support is a service that should be offered to all clients wanting to stop smoking, and smoking cessation interventions have been shown to be effective in mentally ill clients residing in the community [36]. The reason for the high smoking rates among persons with mental illness may, in part, be related to mental healthcare providers' reluctance to integrate interventions for tobacco reduction into their practice, and the lack of attention given to tobacco dependence in organizations providing services for the mentally ill. Integrated solutions must include preparing mental health providers to support tobacco reduction and smoking cessation efforts.

It is clear that the economic costs of tobacco use place a significant burden on people with serious mental illness, especially because many rely on government subsistence, which is well below the poverty line [37]. At the time of this survey, income from a disability pension was capped at $856.42 per month. Social assistance for a single person with a disability, provided by the Government of BC, was 62% of the low-income cut off established by the federal government [38]. Smokers in this study spent an average of $160 per month on tobacco; almost 20% of their monthly income. In addition, many of the smokers made choices to smoke "butts" and to buy cigarettes instead of food. It is well documented that poverty is associated with poorer health outcomes and the extra burden of tobacco-related effects confounds these people's already compromised health outcomes. Tobacco use treatments have been shown to be highly cost-effective [39]. Subsidizing nicotine replacement therapy (NRT) is efficacious in significantly increasing cessation rates and the number of cessation attempts by smokers wanting to stop smoking [40]. In heavy smokers, higher doses of NRT have been shown to increase cessation rates [41]. A way to reduce both the physical and the economic burden of tobacco is for governments or third-party health insurers to provide nicotine replacement therapeutic products free of charge for people with serious mental illness.

These findings must be considered in light of several methodological limitations. First, the relatively low participation rate limits our ability to generalize to the community-based mental health population as a whole. Other community-based studies of people with mental illness have reported similar response rates [42,43]. There are specific factors associated with seriously mentally ill people's willingness to engage in research [44,45]. Many of these factors affected our ability to recruit participants, including the lack of a supportive research culture in the study settings and a reliance on mental health team staff for client referral. Client-specific factors included a fear that the information provided would not be kept confidential and would have an impact on their healthcare. The length of the questionnaire may have been a barrier; many people believed that they could not complete a 45-minute interview. The presence of some symptoms (e.g., paranoia) may have had an additional impact on recruitment. Another limitation of the study relates to the accuracy of the medical diagnosis data; 19% of the participants did not permit access to their medical records. Our reliance on self-reported diagnosis, for these case, may have resulted in misclassification bias. Additionally, some confidence intervals for the odds ratios were very wide (i.e., cocaine use, being widowed, and having no housing) indicating a lack of precision in these estimates.

## Conclusion

People with serious mental illness have very high rates of tobacco use and levels of nicotine dependence, and bear a significant health and economic burden because of their tobacco use. Many of the factors that are associated with smoking vary by gender, and socio-environmental factors play a key role. Researchers have suggested that smoking, particularly by those with schizophrenia, is likely the result of self-medication for symptoms. Consistent with Srinivasan and Thara's conclusions, we found that social factors, including where one lives, and one's marital status, education, and sources of income are associated with smoking, which suggests a more multifacted explanation of tobacco use in the presence of mental illness is required [46]. The finding that gender is strongly associated with smoking status may be explained by a biological sex-based factor or it may represent further support for the hypothesis that social determinants are significant factors at play. More work must be undertaken to better understand the motivators and reinforcers of tobacco use in this population and to develop appropriate tobacco cessation interventions.

## Competing interests

The authors declare that they have no competing interests.

## Authors' contributions

JLJ was the principal investigator for the study and wrote the major sections of the paper. PAR completed the final data analysis and wrote several components of the paper. LAM assisted with data collection and preliminary analysis and contributed to writing the findings section. CTCO conducted some data analysis. RMP assisted with planning the study and commented on the paper. JLB contributed to the development of the project and offered comments on the paper. MG aided in designing the recruitment strategy and offered comments on the paper. AS and MO were members of the research team and offered comments on the paper. All authors read and approved the final manuscript.

## Pre-publication history

The pre-publication history for this paper can be accessed here:

http://www.biomedcentral.com/1471-244X/10/101/prepub
